# Clinical features and hormonal profiles of cloprostenol-induced early abortions in heifers monitored by ultrasonography

**DOI:** 10.1186/1751-0147-48-23

**Published:** 2006-11-23

**Authors:** Fikre Lobago, Hans Gustafsson, Merga Bekana, Jean-François Beckers, Hans Kindahl

**Affiliations:** 1 Department of Clinical Sciences, Faculty of Veterinary Medicine and Animal Science, Swedish University of Agricultural Sciences, Box 7054, SE-750 07, Uppsala, Sweden; 2 Swedish Dairy Association, SE-63184, Uppsala, Sweden; 3 Faculty of Veterinary Medicine, Addis Ababa University, P.O. Box 34, Debre Zeit, Ethiopia; 4 Department of Physiology of Animal Reproduction, Faculty of Veterinary Medicine, University of Liège, Liège, Belgium

## Abstract

**Background:**

The present study describes the clinical features and plasma profiles of bovine pregnancy-associated glycoprotein 1 (bPAG1), the main metabolite of prostaglandin F_2α _(PG metabolite) and progesterone (P4) in heifers in which early abortions were induced.

**Methods:**

Early abortions were induced in four heifers with cloprostenol and monitored by ultrasonography. Blood samples were collected and the plasma were analyzed for bPAG 1, P4 and PG metabolite.

**Results:**

The foetal heartbeat rates varied from 170–186 beats per minute for all foetuses up to the date of cloprostenol treatment. Foetal death was confirmed within two days after cloprostenol treatment. Prior to cloprostenol injection, blood plasma concentrations of bPAG1, PG metabolite and P4 varied from 8.4 – 40.0 ng/mL, 158 – 275 pmol/L and 20.7 – 46.9 nmol/L, respectively. After the foetus expelled, the plasma level of bPAG1 began to decrease but the decrease was small and gradual. The estimated half-life of bPAG1 was 1.8 – 6.6 days. The plasma level of the PG metabolite started to have short lasting peaks (above 300 pmol/L) within three hours after cloprostenol treatment. The plasma concentrations of P4 dropped sharply to less than 4 nmol/L after 24 hours of cloprostenol injection.

**Conclusion:**

The current findings indicated that after early closprostenol-induced foetal death, the plasma concentration of bPAG1 decreased gradually and showed a tendency of variation with the stages of pregnancy.

## Background

The isolation and characterization of pregnancy-specific protein B (PSPB) [1] or bovine pregnancy-associated glycoprotein 1 (bPAG1)[2] in the cow by immunoelectrophoresis and the subsequent development of RIA techniques for this protein (for PSPB[3] and for bPAG1[4]) enabled hormonal diagnosis of pregnancy in cattle. Detection of PSPB/bPAG1 in the maternal blood can be a good indicator of pregnancy and foeto-placental viability [5,6]. PSPB/bPAG1 is detected at around 24 days post conception and reaches a peak at approximately the time of parturition and drops after calving but it is detectable for up to 3 months in postpartum cows [3,4,7].

In early (between 30–50 days of pregnancy) abortions induced both by experimental *Arcanobacterium pyogens *infection and by natural prostaglandin F_2α_, the plasma concentration of PSPB fell steadily from the day of inoculation/treatment but the levels remained above the threshold with a half-life time of 7 days. In terms of detecting embryonic loss following infection, monitoring PSPB on a regular basis has advantages over the assessment of progesterone concentration [5]. Thus PSPB/bPAG1 analysis could be used as an alternative test to determine pregnancy after 30 days post breeding but its relative long half-life (7–8 days, [5,8]), imposes limitations especially in post partum cows and in cows after embryonic/foetal mortality.

In cloprostenol induced early abortions in heifers, the clinical features and patterns of prostaglandin F_2α _metabolite (PG metabolite) were categorized into two: after 100 days and before 75 days of pregnancy stages [9]. The heifers in the former group had retained foetal membranes and delayed return to cyclicity and heat whereas heifers in the latter group expelled their foetuses with intact foetal membranes and showed standing heat within three days after injection. Although most pregnancy failures occur due to embryonic/early foetal mortality up to 50 days post breeding, foetal mortality occurs to some extent until 90 days of pregnancy [10,11]. Detailed information about clinical features and plasma profiles of PSPB/bPAG1 and PG metabolite after induced or spontaneous foetal mortality for pregnancy stages after 60 days (for PSPB/bPAG1) and between 75–90 days (for PG metabolite) in cattle is sparse.

Accurate pregnancy diagnosis could be achieved based on the recognition of a proper embryo with a beating heart, between 26 and 34 days, by use of ultrasonography in cattle [12]. Moreover, embryonic mortality could be estimated at an earlier stage by ultrasound investigation than by PSPB or progesterone assays [5,13]. Thus ultrasound could be used to monitor embryonic/foetal viability and consequently to determine the time of embryo/foetal mortality in induced or spontaneous abortions in cattle. Therefore, the present study describes the clinical features and plasma profiles of bPAG1, PG metabolite and progesterone in heifers after cloprostenol induced early abortions (between 60 and 120 days of pregnancy) monitored by ultrasonography.

## Methods

### Experimental animals

Four Swedish Red and White Breed (SRB) heifers (numbered 1–4) were used. The first three heifers were 24 months old while the fourth one was 21 months old at the beginning of the experiment. They were fed according to the Swedish standard and their rectal temperature and clinical status were regularly checked. The experiment was done, with the approval of the local ethical committee, at the Department of Clinical Sciences, Swedish University of Agricultural Sciences, Uppsala.

### Experimental protocol

Heifers were inseminated following standing heat. Heifer-4 conceived on the first insemination whereas heifers 2 and 3 on the second and heifer-1 on the third consecutive inseminations. A transrectal ultrasonography (5 MHz linear array transducer; Aloka SSD-210 DXII, Aloka Co., Tokyo, Japan) was used to confirm pregnancy and monitor embryo/foetal viability. Embryo/foetus viability was monitored once weekly until 7 days before cloprostenol treatment and then daily till foetal death/abortion occurred. Heartbeat rate was determined by counting the number of heartbeats from the video tape recording of the ultrasonographic examinations as described previously [14]. The heifers were given one intramuscular injection of 500 μg of a prostaglandin analogue (cloprostenol, Estrumate^®^, Schering-Plough, Stockholm, Sweden) to induce luteolysis and subsequent foetal death and abortion. At the time of cloprostenol treatment, heifers 1, 2, 3 and 4 were at pregnancy stages of 63, 77, 83 and 116 days, respectively.

Blood samples were collected from the jugular vein starting one week before cloprostenol treatment. The collection was performed by venipuncture every day for the first five days followed by every three hours from two days before until five days post cloprostenol treatment. Two further blood samples were collected on the 7^th ^and 9^th ^days post cloprostenol injection. The blood samples were drawn into heparinized vacuum tubes and centrifuged immediately. The plasma was removed and stored at -20°C until analysed.

### Hormone analysis

Those plasma samples collected two days before and fives days post cloprostenol treatment were analysed for PG while the daily (the first five days and the last two days) and every six hours (two days before and five days post cloprostenol treatment) plasma samples were analysed for P4 and bPAG1. The Plasma samples were analyzed for concentrations of progesterone [15] and PG metabolite [16] according to the radioimmunoassay methods previously described. Whereas bPAG1 analysis was done following the techniques initially described [4] with little modification as described briefly hereunder. As an assay buffer 25 mM Tris HCl, pH 7.6 + 0.1% bovine serum albumin was used throughout the procedure. Bovine ^125^I-PAG labelled according to the chloramine T method [17] was used as a tracer. Antiserum raised in rabbit against bPAG1 was used as the first antibody at an initial dilution of 1:150,000 whereas double antibody precipitation system was used to separate the bound complex.

For the standard curve lyophilized bPAG1 was diluted with assay buffer to get concentrations ranging from 0.78 to 100 ng/mL in a non-preincubated system. Of each standard concentration, 0.1 mL was added to duplicate tubes and diluted with 0.2 mL assay buffer and for the zero standards (B_0_) and non-specific binding (NSB), only 0.3 and 0.4 mL of buffer, respectively was added. Bovine PAG free serum (0.1 mL) was added to all standard curve tubes. For the test plasma samples, duplicate tubes were labelled for each sample and 0.3 mL assay buffer was added to each tube including two more duplicate tubes for quality control. Then 0.1 mL of each test plasma sample and the two quality control sera was added to the respective duplicate tubes. Following this, 0.1 mL of tracer and first antibody were consecutively added to all tubes (except the first antibody for non-specific binding tubes) and mixed gently and incubated overnight at room temperature. A tracer (0.1 mL) alone was added to duplicate tubes for total count (T). The second day, 1 mL of the double antibody precipitation system was added to all tubes except the T and incubated for further 30 minutes. After dilution with 2 mL of assay buffer, all tubes were centrifuged at 1500 × g for 20 minutes and the radioactivity of the pellet (discarding the supernatants) was counted by a gamma counter (LKB Wallac 1261; Wallac Turku, Finland) with a counting efficiency of 75%. The binding ratio of the radiolabelled ^125^I-PAG to the antiserum was considered as 100% in the zero standard (B_0_) assay tube.

The sensitivity of the bPAG1 RIA was 1 ng/mL for the non-preincubated system used. The intra-assay CV for two serum samples with known bPAG1 concentrations (mean ± SD = 2.5 ± 0.3 and 29.3 ± 2.3 ng/mL) each carried out twenty times were 10.8% and 8.2%, respectively. While the inter-assay CV of low and high concentrations of bPAG1 quality control serum pools (mean ± SD = 9.2 ± 0.4 and 50.3 ± 3.0 ng/mL) were 4.5 and 6.0%, respectively.

### Determination of half-life for bPAG1

Half-life for bPAG1 was estimated for the first three heifers and the fourth one starting from 2.3 and 4 days post cloprostenol injection, respectively, at which the fall in plasma bPAG1 concentration was commenced. The following formula was employed [8,18] for the estimation. T_1/2 _= [ln(C/.5C)]/λ

Where C is the plasma concentration of bPAG1 at time zero, and λ is the slope of the regression equation.

## Results

### Clinical and ultrasonographic features

Pregnancy was confirmed between 33 and 40 days post-insemination by detection of heartbeat of the embryo. The foetal heartbeat rates varied from 170–186 beats per minute for all foetuses up to the date of cloprostenol treatment. Foetal death (loss of heartbeat) was confirmed within two days after cloprostenol treatment. Two of the heifers (nos. 1 & 2) had thick mucous vaginal discharge whereas the other two had blood tinged discharge on the second day post cloprostenol treatment. The dead foetuses were expelled within four days after cloprostenol treatment in three of the heifers whereas the fourth was trapped in the vagina and removed manually. There was no grossly visible abnormality in aborted foetuses at necropsy examination. Standing oestrus was observed in two of the heifers (nos. 1 & 2) within two days after abortion. The detailed clinical and ultrasonographic features associated with cloprostenol induced early abortions are summarised in Table 1.

**Table 1:** Clinical and ultrasonographic features associated with cloprostenol induced early abortions in four Swedish Red and White heifers.

**Clinical/Ultrasonographic Features**	**Heifer-1**	**Heifer-2**	**Heifer-3**	**Heifer-4**
Stages of gestation* (days)	63	77	83	116
Heartbeat rate (beats per minute)	170–182	178–186	180–184	176–182
Foetal death post-CP treatment (Hrs)	< 24	< 24	24 – 48	24 – 48
Foetus/FM expelled** (Hrs)	56 – 72	80 – 96	75	80 – 96#
CRL of expelled foetus (cm)	9	12	15	30
Foetus expelled with unruptured FM	Yes	Yes	Yes	No#
Thick mucous vaginal discharge	Yes	Yes	No	No
Blood tinged vaginal discharge	No	No	Yes	Yes
Showed standing heat***	Yes	Yes	No	No

*on the day of cloprostenol treatment; **after cloprostenol treatment; ***within two days after abortion; #only the foetal membrane was expelled but the foetus was stacked in the vagina and removed manually; CP = cloprostenol; Hrs = hours; CRL = crown-rump length; FM = foetal membrane.

### Plasma hormonal profiles

During one week prior to cloprostenol injection, the plasma concentrations of bPAG1, PG metabolite and progesterone varied from 8.4 – 40.0 ng/mL, 158 – 275 pmol/L and 20.7 – 46.9 nmol/L, respectively. The plasma bPAG1 concentrations were progressively increasing with minor irregularities in heifers 1 and 3 while the changes were irregular in the other two heifers during the one-week period of blood sampled until cloprostenol treatment. Between cloprostenol treatment and expulsion of foetus, the plasma bPAG1 level did not fall but showed minor changes. After foetal expulsion, the plasma level of bPAG1 began to decrease but the decrease was small and gradual up to the last blood sample analysed (6–7 days after foetal expulsion). This decrease in plasma concentrations of bPAG1 was 65.3%, 57.5%, 55.3% and 45.6% for heifers 1–4, respectively. The rate of decrease seems relatively slower with increasing pregnancy stage at the time of cloprostenol injection. On the basis of gradual decline of the plasma levels of bPAG1, a half-life ranging from 1.8 – 6.6 days was estimated.

**Figure 1:**
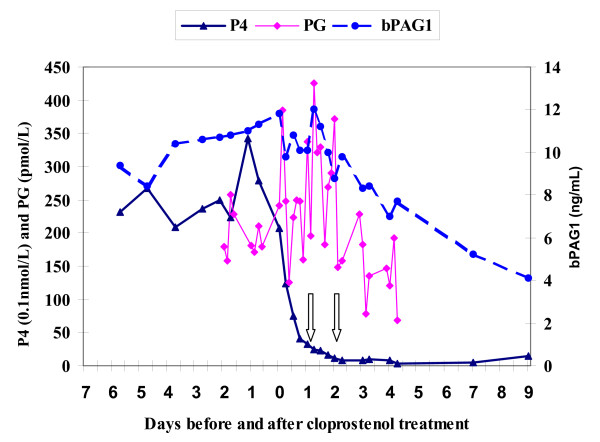
**Plasma profiles of bPAG1, P4 & PG for Heifer-1 before and after cloprostenol treatment on day 0 (63 days post insemination). Foetal death occurred between the two arrows**. P4 = progesterone; bPAG1 = bovine pregnancy associated glycoprotein 1; PG = the main metabolite of prostaglandin F_2α_

**Figure 2:**
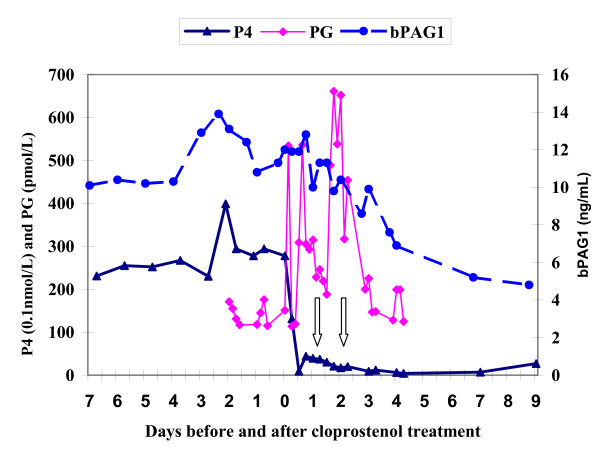
**Plasma profiles of bPAG1, P4 & PG for Heifer-2 before and after cloprostenol treatment on day 0 (77 days post insemination). Foetal death occurred between the two arrows**. P4 = progesterone; bPAG1 = bovine pregnancy associated glycoprotein 1; PG = the main metabolite of prostaglandin F_2α_

**Figure 3:**
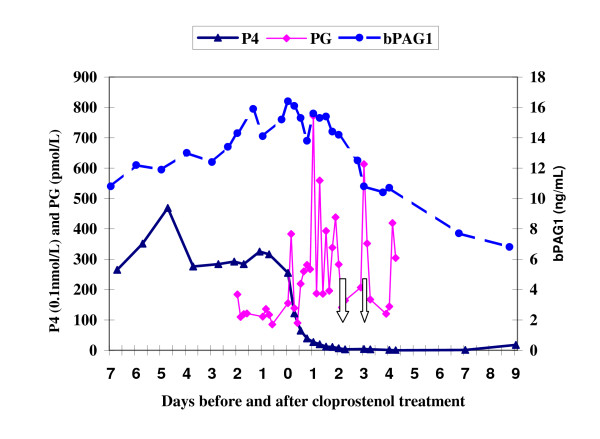
**Plasma profiles of bPAG1, P4 & PG for Heifer-3 before and after cloprostenol treatment on day 0 (83 days post insemination). Foetal death occurred between the two arrows**. P4 = progesterone; bPAG1 = bovine pregnancy associated glycoprotein 1; PG = the main metabolite of prostaglandin F_2α_

**Figure 4:**
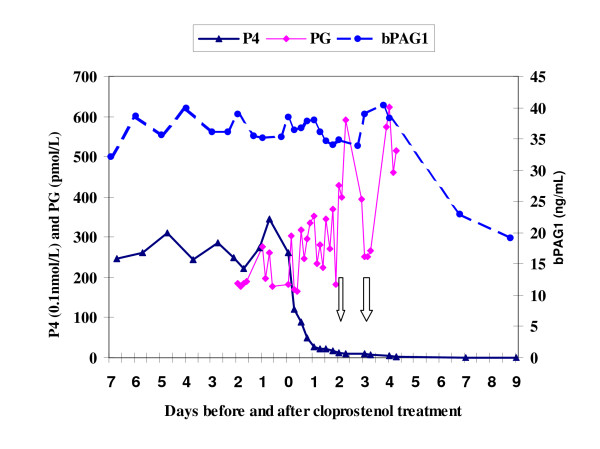
**Plasma profiles of bPAG1, P4 & PG for Heifer-4 before and after cloprostenol treatment on day 0 (116 days post insemination). Foetal death occurred between the arrows**. P4 = progesterone; bPAG1 = bovine pregnancy associated glycoprotein 1; PG = the main metabolite of prostaglandin F_2α_

The plasma level of PG metabolite started to have short lasting peaks (above 300 pmol/L) within three hours after cloprostenol treatment. Then it returned to the pre-treatment level after foetal mortality in the first two heifers (see Fig. 1 &2) but the pulsatile release (above 300 pmol/L) of PG metabolite continued up to the last blood sample analysed (5^th ^day post cloprostenol injection) in the other two heifers especially in the fourth heifer (see Fig. 3 &4).

The plasma concentration of progesterone dropped sharply after the cloprostenol injection and was less than 4 nmol/L after 24 hours in all heifers. From the last plasma sample analysed, the progesterone concentration seems to rise again in the first three heifers (see Fig. 1, 2, 3) indicating resumption of ovarian cyclicity but it remained below detection in the fourth heifer (see Fig. 4).

## Discussion

Because of the unpredictable occurrence of embryonic or foetal losses in a herd and the current increasing interest of monitoring pregnancies in bovine, it is important to monitor pregnancies at early stage. One way to create a model of foetal death is to use drugs like cloprostenol that induce mortality.

Lindell and co-workers [9] induced abortions in heifers and found differences on physical nature of the abortions and PG metabolite release between two different stages of pregnancies (pregnancies below 75 days and between 100 – 150 days). In the former group foetuses were delivered with intact foetal membranes with little or no bleeding whereas in the latter group the aborted foetuses were delivered prior to the membranes, which were retained for more than 24 hours. The proposed reason for the differences in the nature of the induced abortions was the differences in the degree of foetal membrane attachment. The findings of the present study are in agreement with the report of Lindell and co-workers [9], but heifer no. 3 is obviously falling between the two groups since she had blood tinged vaginal discharge and expelled foetus with intact foetal membrane.

Standing oestrus was observed in two of the heifers (nos. 1 & 2) within two days after abortion supporting the suggestion given by Lindell and co-workers [9]. These authors suggested that abortion could be induced up to 80 days of pregnancy for practical reasons with little or no compromise at the subsequent reproductive performance at least in heifers. This is because such abortions are associated with only little or no uterine trauma. This idea is further supported by the return of PG metabolite release to the basal level immediately after abortion in these heifers unlike those heifers above 100 days of pregnancy as it was observed by Lindell and co-workers [9] and the current study.

Detection of PSPB or bPAG1 above the threshold levels in the maternal blood of cows or heifers is a good indicator of the presence of a live embryo or foetus with the exceptions during the postpartum period or for few days after embryonic/foetal death [5,6]. Moreover, the plasma/serum levels of PSPB/bPAG1 fell steadily commencing within 24 hours of inoculation/injection [5] or embryonic/foetal mortality [18] following experimental *Arcanobacterium pyogens *infection or cloprostenol injection. On the other hand, in the current study the gradual fall of plasma bPAG1 concentration commenced after 48 hours of cloprostenol treatment (after expulsion of the foetuses) in three of the heifers and even later in the fourth heifer. The previous experiments involved heifers/cows at pregnancy stages less than 50 days whereas the current one above 60 days, which may explain the observed difference in the time of start of decline in plasma bPAG1 concentration. Szenci and co-workers [18] reported a half-life of 3.2 – 3.9 days of bPAG1 after cloprostenol induced embryonic mortality in heifers, which falls within the range of the current finding. Moreover, Semambo and co-workers [5] reported approximately seven days half-life of PSPB, which is roughly closer to the present finding. The minor differences observed in the half-life of bPAG1 among the reports of different workers may be due to differences in the stages of pregnancy at the time of induction of embryonic/foetal mortality. In the current study the plasma level of bPAG1 did not fall immediately after death of the foetus but it showed minor changes until the foetus was expelled. These minor changes in plasma bPAG1 concentration could be possibly explained by the effect of uterine contraction caused by the pulsatile release of the endogenous PGF_2α _(measured as the metabolite) and a continuity of the placental release of bPAG1 for a brief time even after foetal death. Moreover, the differences in the rate of decline of plasma bPAG1 concentration observed among the heifers at different stages of gestation period could also be possibly attributed to the increase in the plasma concentration and half-life of the glycoprotein with increasing stage of pregnancy.

The plasma PG metabolite level before cloprostenol treatment was in the basal level (below 300 pmol/L) but the level increased immediately after the treatment, which agrees with the results of Lindell and co-workers [9]. However, the reason behind such immediate rise of the peripheral blood level of endogenous PG metabolite after cloprostenol injection is not well established and it needs further investigation.

In heifer no. 4, the pulsatile release of PG metabolite continued up to five days post PG injection even after expulsion of the foetus though the highest concentration of PG metabolite was 623 pmol/L. This plasma PG metabolite concentration is low as compared to the previous report of Lindell and co-workers [9], who reported massive release of PG metabolite up to 2500 pmol/L in heifers having the same pregnancy stage (heifer no. 4) at the time of abortion induced by cloprostenol. This difference in the level of PG metabolite could be partly attributed to the effect of retained foetal membranes in case of the latter heifer with higher level of PG metabolite. This is because cows with retained foetal membranes had significantly higher levels of PG metabolite than cows without retained foetal membranes during the immediate postpartum period [19].

In the current study, a sharp decline of plasma levels of progesterone (from above 20.7 nmol/L to less than 4 nmol/L) during 24 hours post cloprostenol injection was observed. This finding agrees with previous reports that indicated the luteolytic effect of cloprostenol in cyclic non-pregnant or pregnant cows/heifers [9,20-22]. In the present study, the disruption of foeto-endometrial connection as a result of contraction of the uterus caused by the pulsatile release of the endogenous PG metabolite could be the possible cause for the occurrence of foetal deaths within 24 to 48 hours after cloprostenol injection. Szenci and co-workers [18] reported the occurrence of late embryonic mortalities within 24 and between 48 and 72 hours post *Arcanobacterium pyogens *inoculation and cloprostenol treatment, respectively. In another cloprostenol induced abortion study, the progesterone concentration dramatically declined to < 0.5 ng/mL within 24 hours of treatment [5].

## Conclusion

In conclusion, the results of the present study indicated that after early closprostenol-induced foetal death (at pregnancy stages between 60 and 120 days) the plasma concentration of bPAG1 decreased gradually and the rate of decrease showed a tendency of variation with the stages of pregnancy, which requires further confirmation. Moreover, the current finding supports the suggestion [9] that abortion could be successfully induced up to 80 days of pregnancy for practical reasons at least in heifers with little or no compromise to the subsequent reproductive performance.

## Competing interests

The author(s) declare that they have no competing interests.

## Authors' contributions

FL did the blood sampling, clinical and ultrasonographic examinations, laboratory analyses of samples, drafting and revision of the manuscript and participated in the planning of the experiment. HG participated in the planning and coordination of the experiment. MB took part in the planning of the experiment. JFB supervised the laboratory analyses of samples. HK participated in the planning and coordination of the experiment and supervised the laboratory analyses of samples. HG, MB, JFB and HK participated in the critical revision of the manuscript. All authors read and approved the final manuscript.
